# Differential effects of Ydj1 and Sis1 on Hsp70-mediated clearance of stress granules in *Saccharomyces cerevisiae*

**DOI:** 10.1261/rna.053116.115

**Published:** 2015-09

**Authors:** Robert W. Walters, Denise Muhlrad, Jennifer Garcia, Roy Parker

**Affiliations:** 1 Department of Chemistry and Biochemistry, University of Colorado at Boulder, Boulder, Colorado 80303, USA; 2 Howard Hughes Medical Institute, Boulder, Colorado 80303, USA

**Keywords:** stress granules, Hsp70, Sis1, Ydj1

## Abstract

Stress granules and P-bodies are conserved assemblies of nontranslating mRNAs in eukaryotic cells that can be related to RNA–protein aggregates found in some neurodegenerative diseases. Herein, we examine how the Hsp70/Hsp40 protein chaperones affected the assembly and disassembly of stress granules and P-bodies in yeast. We observed that Hsp70 and the Ydj1 and Sis1 Hsp40 proteins accumulated in stress granules and defects in these proteins led to decreases in the disassembly and/or clearance of stress granules. We observed that individual Hsp40 proteins have different effects on stress granules with defects in Ydj1 leading to accumulation of stress granules in the vacuole and limited recovery of translation following stress, which suggests that Ydj1 promotes disassembly of stress granules to promote translation. In contrast, defects in Sis1 did not affect recovery of translation, accumulated cytoplasmic stress granules, and showed reductions in the targeting of stress granules to the vacuole. This demonstrates a new principle whereby alternative disassembly machineries lead to different fates of components within stress granules, thereby providing additional avenues for regulation of their assembly, composition, and function. Moreover, a role for Hsp70 and Hsp40 proteins in stress granule disassembly couples the assembly of these stress responsive structures to the proteostatic state of the cell.

## INTRODUCTION

An emerging aspect of cytoplasmic mRNA biology is the formation of P-bodies and stress granules, which aggregate nontranslating mRNAs and RNA-binding proteins into large mRNP granules. P-bodies contain translationally repressed mRNAs, translational repressors, and components of the mRNA degradation machinery (Stoecklin and Kedersha 2013). mRNAs within P-bodies can be degraded, stored, or can return to translation (Brengues et al. 2005; Bhattacharyya et al. 2006; Parker and Sheth 2007). Stress granules contain nontranslating mRNAs, mRNA-binding proteins, and components of the translation initiation machinery and have been suggested to represent mRNAs that are stalled in the process of translation initiation and as such have partially assembled translation complexes (Buchan 2014; Anderson et al. 2015). Consistent with this view, stress granules are typically observed after treatments, such as stress, which inhibit a step in translation initiation. Stress granules and P-bodies are conserved across eukaryotes and their components play important roles in the control of translation and mRNA degradation (Decker and Parker 2012).

Stress granules and P-bodies are representative of an emerging class of dynamic and nonmembrane bound organelles, although the interactions and cellular complexes that control their assembly and disassembly are not fully understood. Genetic experiments suggest yeast P-body assembly is driven in part by protein–protein interactions, such as the carboxy-terminal domains of Edc3 and Lsm4 (Decker et al. 2007). Several observations suggest that mRNP granule assembly is promoted by intrinsically disordered domains of proteins, which also have predicted tendencies to form β-amyloid structures. First, the prion-related domain of Lsm4 promotes P-body assembly in yeast (Decker et al. 2007; Reijns et al. 2008), and the carboxy-terminal prion domain of TIA1 has been suggested to influence stress granule assembly in mammalian cells (Gilks et al. 2004). Several reports have noted the enrichment of prion-related domains, and so-called low-complexity domains, in RNA-binding proteins and/or components of stress granules and P-bodies (Decker et al. 2007; Reijns et al. 2008; Kato et al. 2012). In several cases, the prion-related domains on RNA-binding proteins have been shown to form homotypic and heterotypic higher order assemblies in vitro (Alberti et al. 2009; Guo et al. 2011; Kato et al. 2012). This has led to a model whereby the accumulation of homotypic and heterotypic interactions between domains of RNA-binding proteins leads to the assembly of mRNP granules, although the nature of any such interactions and their modulation remains unknown.

One possibility is that protein–protein interactions that drive mRNP granule assembly would be modulated by protein chaperones. One abundant class of protein chaperones are the *HSP70* and *HSP40* family members. Hsp70 and Hsp40 proteins act on various states of protein folds (unfolded, misfolded, aggregated, properly folded) to enact distinct outcomes for each of these substrates (Kampinga and Craig 2010). Importantly, Hsp70 and Hsp40 proteins do not act solely on aggregated or misfolded clients, but also actively disassemble cellular structures (Ungewickell et al. 1995; Xing et al. 2010). Moreover, there are increasing examples of HSP40:client interactions that result in outcomes other than refolding or degradation. For example, in *Saccharomyces cerevisiae*, JJJ1 participates in the formation of the 60S ribosomal subunit (Demoinet et al. 2007; Meyer et al. 2007). Broadly, HSP40 family members substantially increase the intrinsically weak ATPase activity of HSP70 and also confer substrate specificity.

Collectively, this raised the possibility that interactions necessary for stress granule or P-body formation would be modulated by chaperones, specifically, HSP70 and HSP40 proteins. Moreover, work in mammalian cells had shown that overexpression of Hsp70 proteins, either due to their induction or by transfection, could limit stress granule formation (Gilks et al. 2004; Mazroui et al. 2007). To examine whether Hsp70 and Hsp40 proteins affected stress granules, we looked at a range of mutants in various *HSP70* and *HSP40* family members in *S. cerevisiae*. We observed that defects in Hsp70 or Hsp40 function inhibited the disassembly/clearance of stress granules during stress recovery. Moreover, various *HSP70* and *HSP40* family members colocalized with stress granules, suggesting they play a direct role in stress granule dynamics. A role for Hsp70 and Hsp40 proteins in stress granule disassembly couples the assembly of these stress responsive structures to the proteostatic state of the cell. We observed that individual Hsp40 proteins have different effects on stress granules. Defects in Ydj1 inhibited recovery of translation following stress and also led to accumulation of stress granules in the vacuole. In contrast, defects in Sis1 did not affect recovery of translation and primarily led to the accumulation of cytoplasmic stress granules. This demonstrates a new principle whereby different disassembly machineries lead to specific fates of components within stress granules, thereby providing additional avenues for regulation of their assembly, composition, and function.

## RESULTS

### Hsp70 function is required for efficient stress granule recovery

To determine how Hsp70 function affects stress granules and P-bodies in yeast, we examined the dynamics of stress granules and P-bodies in strains defective in Hsp70 function. *S. cerevisiae* has four genes for cytoplasmic Hsp70 proteins. Two of these genes are constitutively expressed (*SSA1* and *SSA2*) and two are induced by stress conditions *(SSA3* and *SSA4*) (Werner-Washburne et al. 1987). Since these proteins are largely redundant in their function, we examined stress granules and P-bodies in three strains (Werner-Washburne et al. 1987; Hasin et al. 2014): a wild-type strain, a triple mutant which retains Hsp70 function (*SSA1 ssa2Δ ssa3Δ ssa4Δ*), and a quadruple mutant where the remaining SSA1 gene is compromised with a temperature-sensitive allele (*ssa1-45, ssa2Δ ssa3Δ ssa4Δ*) (Becker et al. 1996; Taxis et al. 2003). To examine stress granules and P-bodies, these strains were transformed with a centromere plasmid expressing Pab1-GFP from its own promoter (a stress granule marker) and Edc3-mCherry from its own promoter (a P-body marker) (Buchan et al. 2008). To avoid complications from cell lethality due to the absence of Hsp70 function, we examined the formation and recovery of stress granules and P-bodies during a stress response at 32°C, where Hsp70 function is compromised specifically in the *ssa1-45*, *ssa2Δ ssa3Δ ssa4Δ* strain, but cells are still viable. This experiment led to several interesting observations.

**FIGURE 1.**
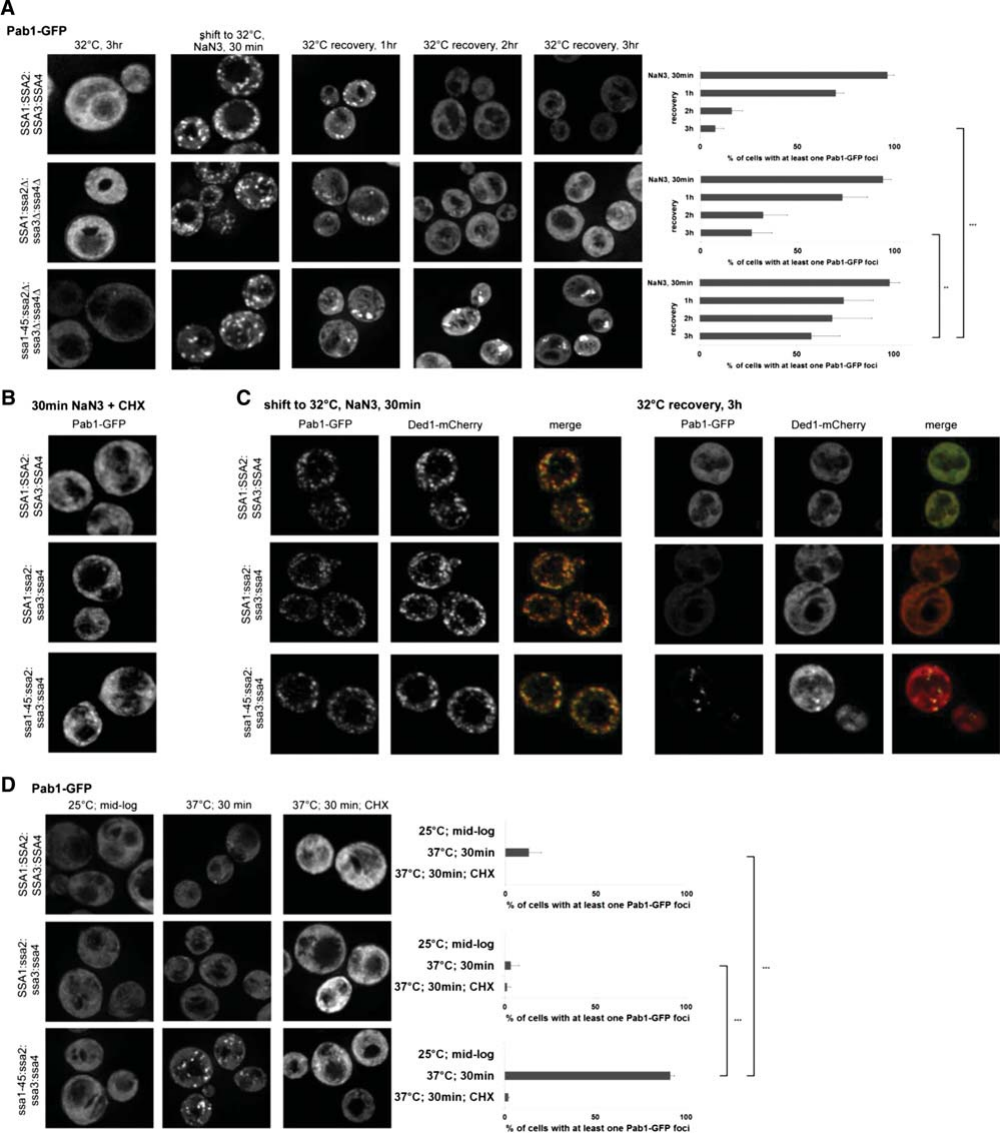
Stress granules persist in *Ssa* defective strains. (*A*) Stress granule induction (NaN3, 30 min) and recovery (removal of NaN3 and addition of fresh media) was analyzed in strains with various combinations of Ssa proteins. Strains were shifted to an intermediate temperature (32°C) for 3 h prior to addition of NaN3 and maintained at 32°C during recovery. Quantification is shown at *right* (percentage of cells with at least one Pab1-GFP foci). (*B*) Foci formation was analyzed in the presence of 100 µg/mL cycloheximide (CHX) (*C*) Pab1-GFP foci colocalize with the stress granule resident protein Ded1-mCherry. (*D*) Total Ssa inhibition results in cycloheximide-sensitive Pab1-GFP foci formation in the absence of stress. The same strains as in *A* were grown at 25°C until mid-log and then shifted to the impermissive temperature 37°C for 30 min and Pab1-GFP localization monitored. All experiments were performed at least three times. Quantification is an average of both multiple images and multiple experimental replicates. Representative images are shown. Error bars represent one standard deviation from the mean. (*) *P* < 0.05, (**) *P* < 0.005, (***) *P* < 0.001.

We observed no increase in Pab1-GFP or Edc3-mCherry foci after a 3 h shift to 32°C in the *ssa1-45*, *ssa2Δ ssa3Δ ssa4Δ* strain without stress (data not shown). This indicates that compromising Hsp70 function to a minimal essential level does not lead to the formation of constitutive stress granules. We also observed that a 30-min treatment of cells with NaN3, which induces stress granules in yeast (Buchan et al. 2011), led to similar inductions of stress granules and P-bodies in all three strains (Fig. 1A and data not shown), indicating that Hsp70 proteins are not required for the induction of stress granules.

An important observation was that strains defective in Hsp70 function showed differences in the rates of stress granule resolution during recovery from stress, wherein NaN3 is removed from the media following a 30-min induction of stress. Specifically, we observed that while Pab1-GFP foci were essentially gone from the wild type or *SSA1 ssa2Δ ssa3Δ ssa4Δ* strains by 2 h, Pab1-GFP foci persisted even out to 3 h and appeared to be enlarged (Fig. 1A). In contrast, P-bodies disassembled normally in the *ssa1-45*, *ssa2Δ ssa3Δ ssa4Δ* strain (data not shown), arguing that Hsp70 proteins do not play a major role in the disassembly/clearance of P-bodies.

Two additional experiments argued that the Pab1-GFP foci persisting in the *ssa1-45*, *ssa2Δ ssa3Δ ssa4Δ* strain were stress granules. First, we observed that the formation of these foci was inhibited by the addition of cycloheximide (Fig. 1B), which traps mRNAs in polysomes and prevents the formation of stress granules (Buchan et al. 2008, 2011). Second, in experiments where we used plasmids expressing Pab1-GFP with Ded1-mCherry or Pub1-mCherry, which are also markers for stress granules, we observed that the Pab1-GFP foci contained Ded1-mCherry and Pub1-mCherry (Fig. 1C and data not shown). These results indicate that when Hsp70 activity is limited, stress granules persist during recovery, which suggests Hsp70 complexes may play a role in disassembly and/or clearance of stress granules. We observed no defect in stress granule formation or clearance in strains individually lacking *Ssa1*, *Ssa2*, *Ssa3*, or *Ssa4* (data not shown), suggesting that different Ssa proteins can affect stress granule dynamics.

Additional evidence that Hsp70 function affects stress granule disassembly or clearance comes from examining Pab1-GFP after a shift of these strains to 37°C. The key observation is that we observed Pab1-GFP foci forming after a 30-min shift to 37°C in the *ssa1-45*, *ssa2Δ ssa3Δ ssa4Δ* strain but not the *SSA1 ssa2Δ ssa3Δ ssa4Δ* or wild-type strains (Fig. 1D). These Pab1-GFP foci were cycloheximide sensitive, suggesting they are stress granules (Fig. 1D). We interpret these foci to be consistent with a model whereby mRNPs are constantly cycling through stress granules but the steady-state concentration is such that stress granules are not visible under the light microscope, and a very strong defect in Hsp70 function leads to the constitutive accumulation of stress granules due to defects in their disassembly and/or clearance.

### Constitutive expression of ATPase-inactive Ssa1 impairs SG recovery

A second way to examine whether Hsp70 proteins affect stress granule recovery is to examine the effect of dominant negative mutations in Hsp70. This approach takes advantage of the Hsp70 ATPase cycle where mutations blocking ATP hydrolysis can dominantly interfere with Hsp70 function (Ha and McKay 1995; Misselwitz et al. 1998; McClellan and Brodsky 2000). Earlier work had shown that overexpression of an ATP hydrolysis mutant, Ssa1-G199D, slowed growth and impaired protein translocation in vivo in a dominant manner (McClellan and Brodsky 2000). Given this, we overexpressed either WT or G199D Ssa1 proteins in yeast and examined their effect on stress granule formation and recovery.

**FIGURE 2.**
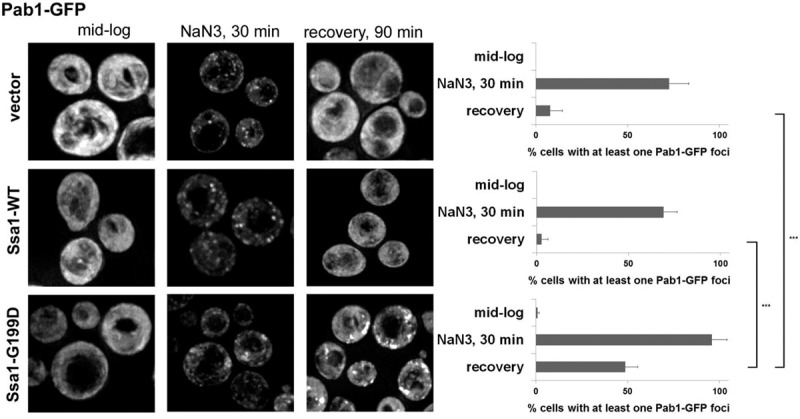
Stress granules persist in the presence of a dominant negative Ssa1 mutant. WT yeast strains were transformed with empty backbone plasmid (*top* panel), WT Ssa1 (*middle* panel), or ATPase-inactive Ssa1 (G199D) (*lower* panel). Cells were grown (all 30°C) to mid-log, stress granules were induced by addition of NaN3 for 30 min and then allowed to recover for 90 min. Cells were scored for Pab1-GFP foci as in Figure 1. Quantification is shown at *right* and performed as in Figure 1. (*) *P* < 0.05, (**) *P* < 0.005, (***) *P* < 0.001.

We did not observe increases in stress granules (as evidenced by Pab1-GFP foci) during mid-log growth or substantial differences between the number of these foci when stress granule formation was induced using NaN3 (Fig. 2). This indicates that expression of Ssa1-G199D was not sufficient to induce constitutive stress granules. More important, we observed that strains expressing the Ssa1-G199D variant failed to disassemble/clear stress granules effectively during stress recovery (Fig. 2). We interpret this observation to argue that failure of Ssa1 to bind substrates effectively leads to an inability to resolve stress granules after stress removal, and provides a second line of evidence that Hsp70 function is required for stress granule resolution.

Given the effects of Hsp70 defects on stress granule disassembly, we examined how other heat shock–induced protein chaperones affected stress granules. We observed that strains lacking *Hsp26*, *Hsp42*, or *Hsp104* did not show any defects in stress granule clearance (Supplemental Fig. S1), indicating that not all protein chaperones affect stress granules.

### Several SSA proteins colocalize with stress granules in yeast

**FIGURE 3.**
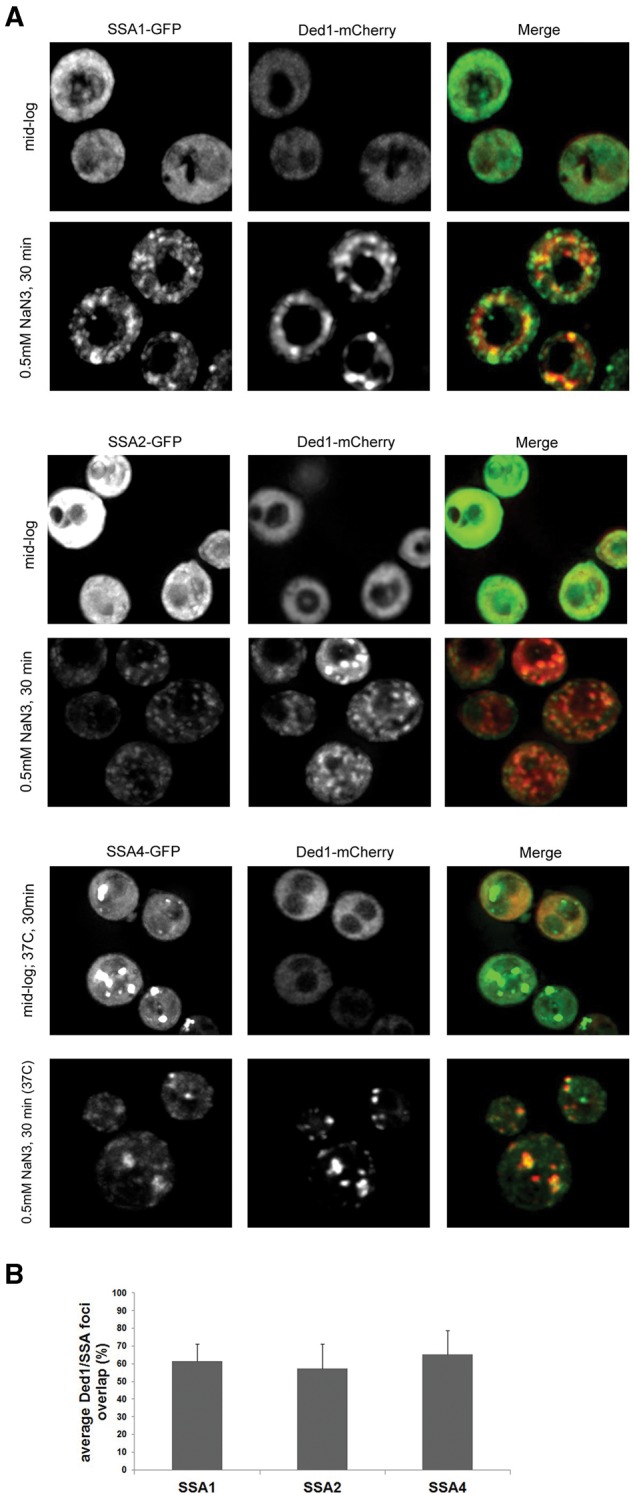
Ssa1, −2, and −4 colocalize with stress granules. (*A*) The indicated GFP-tagged strains (from the yeast GFP collection) were transformed with the stress granule marker Ded1-mCherry. GFP and mCherry overlap was assessed during mid-log growth and after NaN3 treatment for 30 min. Overlap was defined by first identifying Ded1-mCherry foci. At least 100 of these foci (randomly chosen) for each condition and strain tested were then assessed for GFP colocalization. (*B*) Quantification was done as previously and expressed as average Ded1/Ssa foci overlap per cell. The *Ssa4-GFP* strain was first incubated at 37°C to induce Ssa4-GFP expression.

In principle, Hsp70 proteins could be affecting stress granules by direct and/or indirect effects. If Hsp70 proteins directly act on components of stress granules, they are predicted to localize to stress granules. To test this, we transformed GFP-tagged Ssa1, Ssa2, and Ssa4 strains with the stress granule marker, Ded1-mCherry (Fig. 3) and examined if GFP-tagged SSA proteins colocalized with Ded1-mCherry foci. We did not examine Ssa3 localization since there was low GFP expression in the Ssa3-GFP strain.

We observed strong colocalization (∼70% of all Ded1-mCherry foci contained a corresponding Ssa-GFP foci) of Ssa1-GFP and Ssa2-GFP with Ded1-mCherry, consistent with a direct role of these proteins in stress granule dynamics (Fig. 3). We did not observe Ssa4-GFP expression in mid-log cultures or in response to 30 min of NaN3, which is consistent with this gene being induced by heat stress (Werner-Washburne et al. 1987). To examine Ssa4-GFP localization to stress granules, we first heat shocked the cells for 30 min at 37°C, which does not by itself induce stress granules (Buchan et al. 2011; Grousl et al. 2009), followed by treatment with NaN3 to induce stress granules. We observed that Ssa4-GFP also showed colocalization with stress granules under these conditions (Fig. 3). Thus, Hsp70 proteins localize to stress granules and therefore could directly affect protein rearrangements within stress granules.

### Sis1 and Ydj1 colocalize with stress granules

**FIGURE 4.**
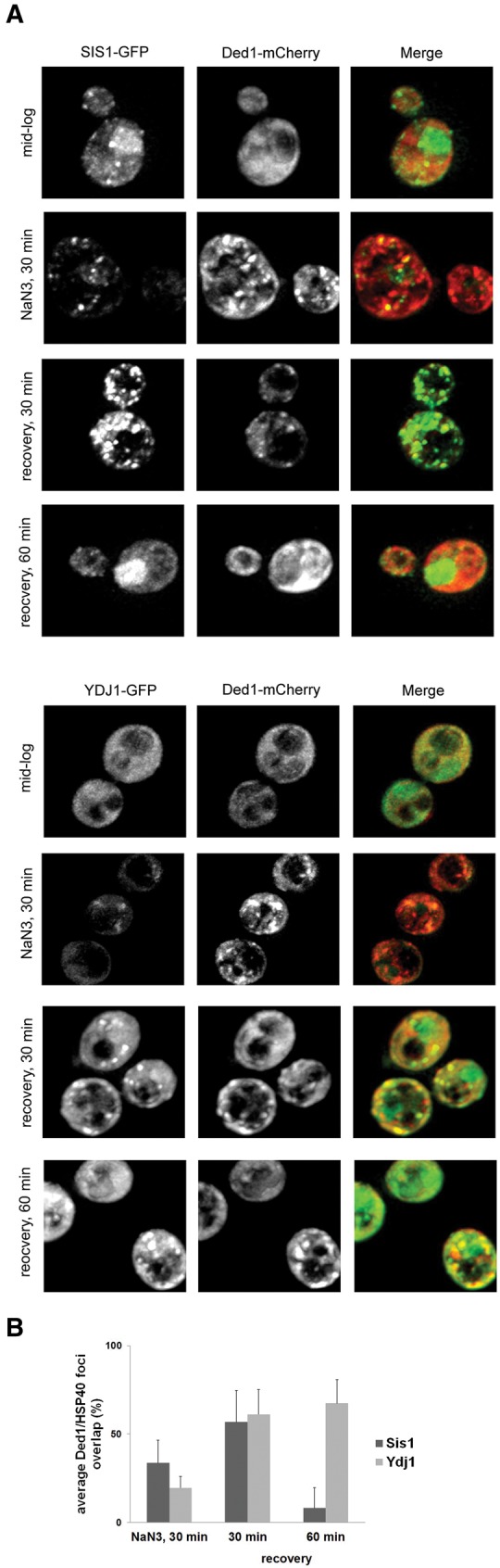
Sis1 and Ydj1 colocalize with stress granules. (*A*) The indicated strains were transformed with the stress granule marker Ded1-mCherry. GFP and mCherry overlap was assessed as in Figure 3 and quantification is presented in *B*.

Hsp70s typically function with Hsp40 proteins that both stimulate the Hsp70 ATPase activity and serve as adaptor proteins to provide substrate specificity for Hsp70 proteins. To examine if specific Hsp40 proteins were directly involved in stress granule dynamics, we screened Hsp40 members tagged for colocalization with a Ded1-mCherry marker under mid-log growth, after stress granule induction, and during recovery. Of the 22 Hsp40s found in the *Saccharomyces* genome, nine gave interpretable GFP signals above background fluorescence (Fig. 4; Supplemental Fig. S2).

We observed that two HSP40 proteins, Ydj1 and Sis1, showed some colocalization with Ded1-mCherry (Fig. 4), although there were some differences in the timing and extent of their association with stress granules. In the absence of stress, Sis1-GFP was primarily nuclear with some heterogeneous cytoplasmic distribution, but after 30 min of NaN3 was dispersed from the nucleus and accumulated in cytoplasmic foci that colocalized with Ded1-mCherry (Fig. 4A, top panels). At 30 min of recovery, Sis1-GFP was still present in stress granules, but by 60′ of recovery, Sis1-GFP had exited stress granules and reaccumulated in the nucleus. In contrast, in the absence of stress Ydj1-GFP was diffusely cytoplasmic, during NaN3 stress accumulated weakly in stress granules (as defined by overlap with Ded1-mCherry, Fig. 4A, lower panels), but showed more robust accumulation in stress granules after 30′ and 60′ of recovery (Fig. 4A, lower panels). The presence of Sis1 and Ydj1 in stress granules suggests that these proteins might affect stress granule assembly and/or disassembly.

### Ydj1 and Sis1 affect stress granule disassembly and subcellular localization

**FIGURE 5.**
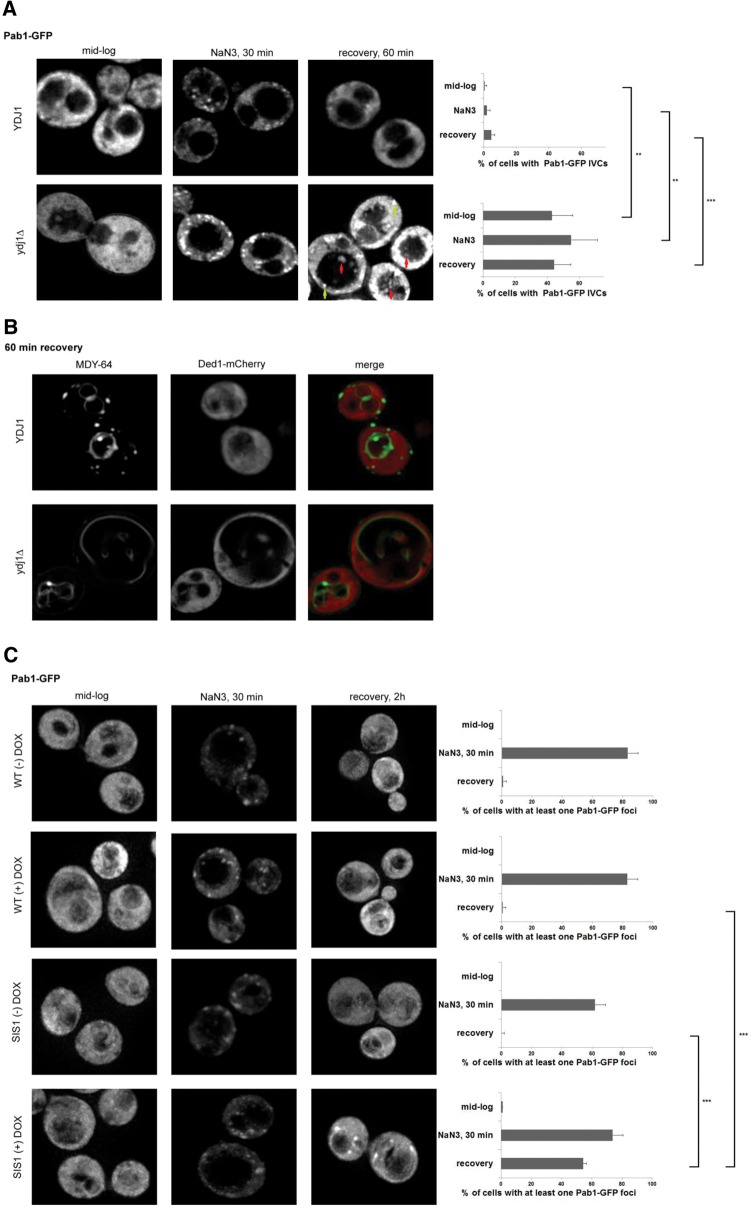
Stress granule recovery defects in *ydj1* and *sis1* defective strains. (*A*) Stress granules in WT (*YDJ1*) and *ydj1Δ* cells were analyzed during mid-log, after 30 min of NaN3 stress, and after 60 min of recovery using Pab1-GFP as a marker. Quantification is shown at *right* and was done as in previous experiments. Representative images are shown; the images displayed are individual *z*-stack images (as opposed to collapsed collections of individual *z* slices). (*B*) Outer vacuolar membranes were labeled with the green fluorescent dye, MDY-64. The same strains were analyzed as in *A*, after 60 min of recovery, but using Ded1-mCherry as the stress granule marker. (*C*) *WT* or *Sis1-Tet-off* strains were grown for ∼18 h either in the presence (+) or absence (−) of 20 µg/mL doxycycline (Park et al. 2013). Cells were back diluted; fresh doxycycline was added (+ only) and grown until mid-log (OD ∼0.5). Pab1-GFP was analyzed as previously during mid-log and after stress granule induction. Quantitation is shown at *right*.

To examine the possible functions of Ydj1 and Sis1 on stress granules, we examined stress granule formation and resolution in strains defective in these proteins. The *ydj1Δ* strain is viable and we observed that *ydj1Δ* strains do not harbor constitutive stress granules during mid-log growth or show differences from wild-type cells in stress granule induction using NaN3 (Fig. 5). However, during recovery, *ydj1Δ* strains showed Pab1-GFP foci accumulation in circular structures within the yeast vacuole for ∼50% of the cells examined (marked with red arrows in Fig. 5A, quantifying next to figure), as well as some persistence of cytoplasmic stress granules (marked with yellow arrows in Fig. 5A). Intravacuolar structures containing stress granules have previously been described in yeast strains lacking the Atg15 lipase, which is involved in the breakdown of intravacuolar autophagic vesicles (IVCs) (Buchan et al. 2013). We also observed that a second stress granule marker, Ded1-mCherry, accumulated in intravacuolar structures in *ydj1Δ* strains, whose periphery stained with the vacuolar dye, MDY-64, which stains vacuolar membranes (Fig. 5B; McKane et al. 2006). We interpret these observations to suggest that Ydj1 plays a role in stress granule dynamics, and in the absence of Ydj1, stress granules accumulate both in the cytosol and in intravacuolar compartments (see Discussion).

We also examined the effects of Sis1 defects on stress granule dynamics. Sis1 is an essential protein; thus we utilized a Tet-off Sis1 strain wherein Sis1 protein is ∼30% reduced by genomic insertion of the Tet-off promoter, and is completely absent after overnight incubation with doxycycline (Fig. 7C, below; Park et al. 2013). A plasmid expressing Pab1-GFP was introduced into the Sis1-Tet-off strain (and corresponding “WT” strain), grown in the presence or absence of doxycycline, and then examined for their response and recovery during NaN3 treatment. Strikingly, we observed the persistence of cytoplasmic stress granules when Sis1 was depleted without the accumulation of stress granule markers within intravacuolar compartments (Fig. 5C). We interpret this observation to suggest that Sis1 is required for efficient stress granule clearance in yeast.

### Ydj1 and Sis1 direct stress granules to different fates: Ydj1 is required for the resumption of translation after stress

The phenotypes of Sis1 and Ydj1 mutations on stress granules suggested they might impact stress granules in different manners. Previous work has suggested that stress granules can either be disassembled for return of mRNAs into translation (Buchan and Parker 2009), or can be targeted for autophagy (Buchan et al. 2013). Given these two fates, we examined how Sis1 and Ydj1 mutations affected these two fates of stress granules.

To examine how Sis1 and Ydj1 affected the ability of mRNAs within stress granules to return to translation, we examined the effects of Sis1 and Ydj1 mutations on global translation during mid-log growth, during a stress response, and during recovery. For this experiment, wild-type, *ydj1Δ*, and Sis1-tet-off strains were pulse-labeled for 5′ at various times and the labeled proteins examined by SDS-PAGE.

**FIGURE 6.**
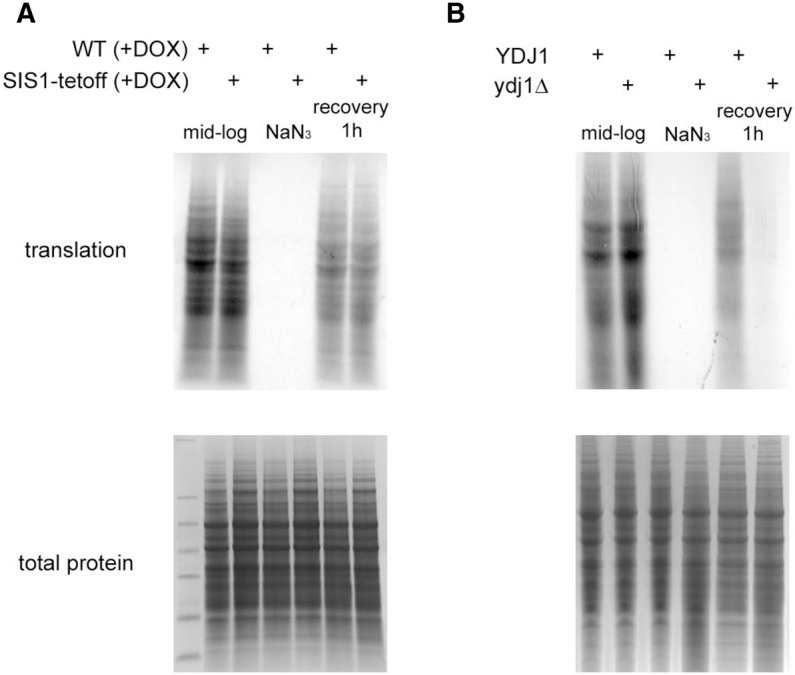
Cells lacking Ydj1 fail to resume translation during stress recovery. Strains were labeled for 5′ with S35 amino acids either in mid-log, after 30′ of NaN3-induced stress, or after 60′ of stress recovery. (*A*) *Tet-off Sis1* compared to wild-type control. (*B*) *ydj1Δ* compared to wild-type control. For each gel, a Coomassie staining showing equal loading is shown *below* the radioactive image.

We observed that both wild-type and Sis1-Tet-off strains behaved similarly with efficient translation before stress, a strong repression of translation during stress, and the resumption of translation after 1 h of recovery (Fig. 6A). In contrast, the *ydj1Δ* strain is unable to recover translation efficiently from stress (Fig. 6B). We interpret these observations to argue that Ydj1 acts on components of stress granules in a manner that promotes mRNAs within stress granules reentering translation. The efficient resumption of translation in the Tet-off Sis1 suggests that Sis1 is not required for mRNAs to reenter translation and might affect a different path of stress granule clearance.

### Ydj1 and Sis1 direct stress granules to different fates: Sis1 promotes targeting of stress granules to autophagy

As Sis1 did not affect the resumption of translation during stress recovery, we hypothesized that Sis1 might play a role in stress granule clearance by affecting the autophagy of stress granules (Buchan et al. 2013). A prediction of this interpretation is that defects in Sis1 would lead to a decrease in the targeting of stress granules for autophagy. Autophagy of stress granules can easily be observed in *atg15Δ* strains (Buchan et al. 2013), where the intravacuolar compartments accumulate because of the absence of the Atg15 lipase that normally degrades such compartments (Teter et al. 2001). Given this, we determined if Sis1-Tet-off strains showed a decrease in the accumulation of stress granule markers in intravacuolar compartments in *atg15Δ* backgrounds.

**FIGURE 7.**
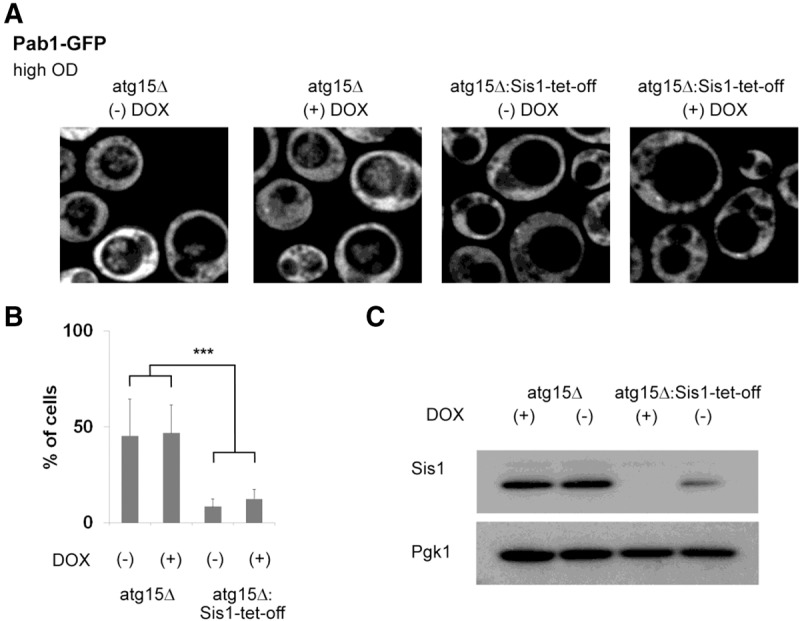
Stress granule vacuolar accumulation is impaired with decreased Sis1 expression. (*A*) Cells were grown to OD600 of ∼2 (∼18 h) in the presence or absence of 20 µg/mL doxycycline and Pab1-GFP accumulation in vacuoles analyzed. As in Figure 6, images shown are individual *z* slices. (*B*) Quantitation is shown and was done as previously. (*) *P* < 0.05, (**) *P* < 0.005, (***) *P* < 0.001. (*C*) Sis1 protein expression Western analysis with Pgk1 Western as a loading control.

Consistent with earlier results, we observed that the *atg15Δ* alone accumulated large numbers of cells with stress granule markers in intravacuolar compartments (Fig. 7). In an *atg15Δ* tet-off Sis1 strain, a substantial reduction of stress granule markers in intravacuolar compartments was observed (Fig. 7). Surprisingly, this effect was similar in the presence or absence of doxycycline. However, we and Park et al. (2013) observed that the Sis1-Tet-off strain shows a ∼30% reduction in Sis1 protein even without doxycycline (Fig. 7C). This result suggests that subtle changes in Sis1 affect stress granule targeting to vacuoles. We interpret these results to suggest that Sis1 promotes the clearance of stress granules by autophagy.

## DISCUSSION

We present several lines of evidence that Hsp70 proteins are involved in the disassembly of yeast stress granules. First, temperature-sensitive strains deficient in Hsp70 function show persistent stress granules during recovery from stress (Fig. 1). Second, overexpression of the dominant negative Ssa1-G199D allele leads to persistence of stress granules during stress recovery (Fig. 2). Third, the Ssa1, Ssa2, and Ssa4 proteins colocalize with stress granules (Fig. 3). Two previous observations argue that Hsp70 proteins also affect stress granule dynamics in mammalian cells. Specifically, overexpression of the prion-related domain of TIA1 induces Hsp70 expression in mammalian cells and can reduce the nuclear aggregation of this isolated protein domain as well as limit stress granule formation (Gilks et al. 2004). Similarly, overexpression of HSP70 family members in mammalian cells in culture can reduce the induction of stress granules by proteasome inhibitors (Mazroui et al. 2007). Although the simplest model is that Hsp70/Hsp40 complexes directly affect stress granules, it remains formally possible that the effects we observe in *hsp70* and *hsp40* mutants are due to indirect effects.

Two main types of observations argue that the Hsp40 members Ydj1 and Sis1 are also involved in stress granule disassembly and/or clearance. First, Ydj1 and Sis1 tagged with GFP colocalize with stress granule markers during NaN3 treatment and subsequent recovery (Fig. 4). Second, yeast strains lacking *Ydj1* or *Sis1* show alterations in stress granule disassembly/clearance during recovery (Fig. 5). A role for both Hsp70 and Hsp40s in stress granule clearance is consistent with these proteins working together to alter the conformation and/or interactions of specific components of stress granules and thereby influence their fates.

**FIGURE 8.**
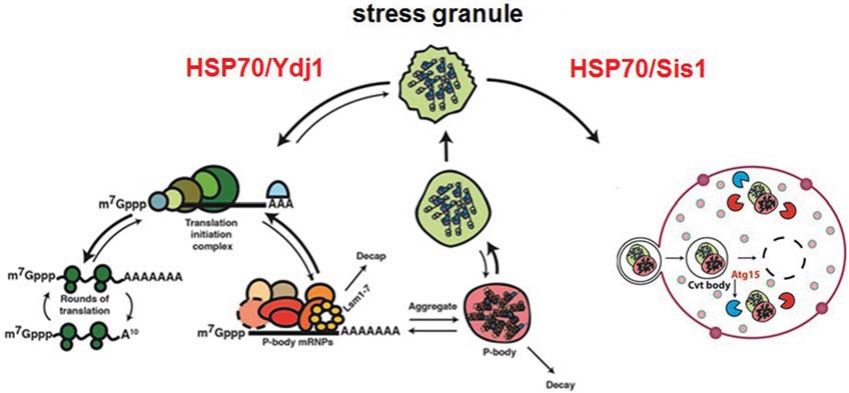
Model for differential effects of Sis1 and Ydj1 on stress granules. Several mRNPs (stress granules, P-bodies, ribosomes, and translation initiation complexes) and cellular structures (vacuoles; *far right*, outlined in red) are depicted. Arrows indicate directionality of mRNP transitions to distinct states.

Our data suggest that Ydj1 primarily affects the disassembly of stress granules in a manner whereby mRNAs can return to translation (depicted in Fig. 8). The key observation is that *ydj1Δ* strains are unable to restore translation effectively during stress recovery (Fig. 6). Such a role of Ydj1 during stress recovery may provide a molecular explanation for why *ydj1Δ* strains are hypersensitive to numerous stress inducers (Outten et al. 2005; Qian et al. 2012). Two models could explain Ydj1's role in the restoration of translation. First, Ydj1 could participate in disassembling a translationally repressed mRNP that accumulates in stress granules, and this disassembly could help to restore translation. Alternatively, Ydj1 could restore function to a defective translation initiation factor during stress recovery and thereby restore translation, which then leads to loss of stress granules.

We also observed that *ydj1Δ* strains accumulated stress granules in the cytosol and in intravacuolar compartments during stress recovery (Fig. 5). We interpret this phenotype to result from a defect in stress granule disassembly (Fig. 8), which then leads to a persistent pool of cytoplasmic stress granules, many of which are then targeted for autophagy leading to a buildup of stress granules within the vacuole.

Two observations suggest that Sis1 affects stress granules in a different manner from Ydj1 and may primarily play a role in targeting stress granules for autophagy (Fig. 8). First, Sis1 is not required for restoring translation during stress recovery (Fig. 6), arguing it does not promote mRNAs reentering translation from stress granules. Moreover, when Sis1 protein levels are reduced, the accumulation of stress granules within the vacuole of an *atg15Δ* strain declined (Fig. 7). An interesting possibility is that “stress granule” material that persists in a *sis1* mutant strain during recovery is a subset of individual stress granules, or components within them, that are normally not disassembled and instead are cleared from the cell by autophagy.

It is likely the different Sis1 and Ydj1 phenotypes are a result of distinct client binding activities within stress granules. Sis1 and Ydj1 have been previously shown to differentially regulate both artificial (denatured luciferase [Lu and Cyr 1998] and prion substrates [Reidy et al. 2014]). Intriguingly, in one such case, the [RNQ+] prion Sis1 bound to the non-prion domain, while Ydj1 bound to the prion domain (Douglas et al. 2008; Summers et al. 2009). Resident stress granule proteins and RNA-binding proteins are enriched in intrinsically disordered domains that are similar to prion-like domains. One hypothesis is that Ydj1 and Sis1 target different classes of such intrinsically disordered domains and thereby lead to different fates for the components of stress granules.

Several observations argue that the structures we observe are bona fide stress granules, as opposed to “heat stress granules” (Cherkasov et al. 2013). First, Pab1-GFP foci in this work are cycloheximide sensitive and contain additional SG markers (Fig. 1). Second, the temperature shifts we use do not typically cause proteins to aggregate or stress granules to form (Nathan et al. 1997; Buchan and Parker 2009; Verghese et al. 2012). Third, we do not observe an HSP104-dependent recovery (Supplemental Fig. S1), as in the case with heat stress granules (Cherkasov et al. 2013). It is possible that in the absence of HSP70/40 proteins, distinct protein aggregates also form, and might even merge with stress granules under some conditions (Buchan et al. 2013).

A role for Hsp70/Hsp40 proteins in stress granule disassembly has three important implications. First, it suggests that assembly of stress granules will be tunable to the proteostatic state of the cell. Specifically, when stress first occurs additional substrates for Hsp70 are generated, which would be expected to reduce the ability of Hsp70 complexes to keep stress granules in a disassembled state, leading to a tendency toward assembly. As chaperones are induced and/or the load of misfolded proteins reduced, unoccupied Hsp70 complexes will increase and thereby be able to participate in stress granule disassembly. The ability of the stress granule assembly mechanism to be tunable to the proteostatic state of the cell may be beneficial for properly modulating translation during stress and thus stress survival.

A second interesting implication is that our observations could explain how mutations in a Sis1-related Hsp40 protein in humans lead to a type of muscle myopathy. Specifically, dominant mutations in the human Hsp40 *DNAJB6* lead to limb-girdle muscular dystrophy, which is a late onset degenerative muscle myopathy (Couthouis et al. 2014). Since hyperformation or persistence of stress granules, or stress granule-related complexes, appear to be causative in some neurodegenerative diseases such as ALS and some muscle myopathies (Li et al. 2013; Ramaswami et al. 2013), one possibility is that these mutations in *DNAJB6* lead to a reduced ability to clear stress granules by autophagy, which could contribute to the degenerative disease.

A third implication of this work is that it adds to a growing number of ATP-driven machines that play roles in modulating the disassembly/clearance of stress granules. In addition to the Hsp70/Hsp40 machines identified here, previous work has indicated that Cdc48/VCP plays a role in targeting stress granules for autophagy, presumably by the extraction of a stress granule component that decreases autophagy (Buchan et al. 2013). Similarly, the ATPase activity of Ded1 has been shown to be required for mRNAs to exit stress granules and return to translation (Hilliker et al. 2011). Taken together, these observations suggest that stress granules are acted on by a number of different ATP-driven machines and that those machines are likely to play an important role in maintaining the assembly/disassembly of these complexes. It should be noted that since multiple different ATPases can act on stress granules, it allows numerous regulatory mechanisms in the cell to control the protein and mRNA composition of stress granules.

## MATERIALS AND METHODS

### Yeast strains and plasmids

yRP2908, 2909, 2912, 2914 were kind gifts of Dr. Elizabeth Craig (University of Wisconsin). yRP2990, 2991, and 2992, and were kind gifts of Dr. Jeffrey Brodsky (University of Pittsburgh). Tet-off strains were obtained from Open Biosystems. Indicated GFP-tagged strains are from the yeast GFP collection. *atg15Δ:Sis1-Tet-off* (yRP3024) and *atg15Δ:Tet-off* (yRP3017) strains were derived using standard genetic methodologies. All strains and plasmids used in this study are listed in Supplemental Table S1.

### Microscopy

Yeast cultures were grown to OD600 of 0.4–0.5 in the appropriate minimal synthetic defined (SD) media. Temperatures are indicated for individual experiments. For stress recovery experiments, cells were collected by brief centrifugation, washed in fresh SD medium, resuspended in fresh SD medium to maintain equal cell density, and incubated at indicated temperature and time. Cells were then spotted on slides and immediately subjected to microscopic examination at room temperature.

All images were acquired using a Deltavision Elite microscope system running softWoRx 6.3 software (Applied Precision, LLC), using an Olympus 100×, oil-immersion 1.4 NA objective. They were collected as 2056 × 2056 pixel files with a cMOS camera (Photometrics) using 1 × 1 binning. All yeast images were deconvolved using standard softWoRx deconvolution algorithms (enhanced ratio, medium noise filtering). ImageJ (Abramoff et al. 2004) was used to adjust all images to equal contrast ranges according to the experiment conducted or protein examined. To optimize yeast colocalization accuracy, single plane images were used, with mCherry imaging immediately after GFP imaging. Images are *Z*-series compilations of 8–10 images per stack; images assessing vacuoles are individual *Z*-stack slices.

### Image quantitation

Quantified data sets involving Pab1-GFP and Ded1-mCherry represent the analysis of at least three independent experiments. All scoring analyses were done in a blind manner, with a minimum of 100 cells scored for stress granule data, and 100 individual granules for colocalization assays. To count and measure foci size, ImageJ smoothing, thresholding, and analyze particle functions were used.

### Vacuolar staining

MDY-64 was obtained from Life Technologies. Briefly, at indicated time points, cells were resuspended at 2–4 OD600 units/mL in labeling buffer (10 mM HEPES, pH 7.4, 5% glucose). MDY-64 was then added to a final concentration of 10 µM and cells were incubated for 5 min at room temperature. Cells were then resuspended in fresh labeling buffer and analyzed by microscopy.

### ^35^S-Met incorporation assays

Cells from wild-type and isogenic deletion strains were grown in SD media (lacking corresponding amino acids) to mid-log phase (OD600 0.3–0.35), and resuspended to equal optical density units. Samples were washed twice in minimal media, followed by resuspension in minimal media, and incubation in a flask placed in a water bath shaker at the indicated temperature for 10 min. Samples were concentrated, resuspended in one-tenth the original volume of SD media lacking methionine, and labeled with ^35^S- Met/Cys for 5 min. Cells were harvested by a quick spin and frozen immediately in dry ice. Proteins were extracted by boiling cell pellets for 10 min in 50 µL lysis buffer (2% SDS, 90 mM HEPES, pH 7.5, and 30 mM DTT), spinning for 2 min in a microfuge, and transferring the supernatant to a new tube. An aliquot of this was combined with denaturing protein loading buffer, run on a 10% SDS-PAGE gel, and imaged on a Typhoon PhosphorImager.

### Western blot analysis

Sis1 antibody was a kind gift of Dr. Douglas Cyr (University of North Carolina). Western blot analysis was carried out using standard methodology and as previously described (Walters et al. 2014).

## SUPPLEMENTAL MATERIAL

Supplemental material is available for this article.

## Supplementary Material

Supplemental Material
