# Supplementary material for: Differential effects of Ydj1 and Sis1 on Hsp70-mediated clearance of stress granules in Saccharomyces cerevisiae
Source: RNA. 2015 Sep;21(9):1660–71. doi: 10.1261/rna.053116.115 (PMC4536325; doi:10.1261/rna.053116.115)
Supplement: Supplemental Material [file supp_21_9_1660__index.html]

Differential effects of Ydj1 and Sis1 on Hsp70-mediated clearance of stress granules in Saccharomyces cerevisiae — Differential effects of Ydj1 and Sis1 on Hsp70-mediated clearance of stress granules in Saccharomyces cerevisiae — Supplemental Material 

# Differential effects of Ydj1 and Sis1 on Hsp70-mediated clearance of stress granules in *Saccharomyces cerevisiae*

## Supplemental Material

**Files in this Data Supplement:**

- Supp Figures & Tables.docx
