# Supplementary material for: Differential effects of Ydj1 and Sis1 on Hsp70-mediated clearance of stress granules in Saccharomyces cerevisiae
Source: RNA. 2015 Sep;21(9):1660–71. doi: 10.1261/rna.053116.115 (PMC4536325; doi:10.1261/rna.053116.115)
Supplement: Supplemental Material [file supp_053116.115_SuppFigures_Tables.docx]

**FIGURE S1.**


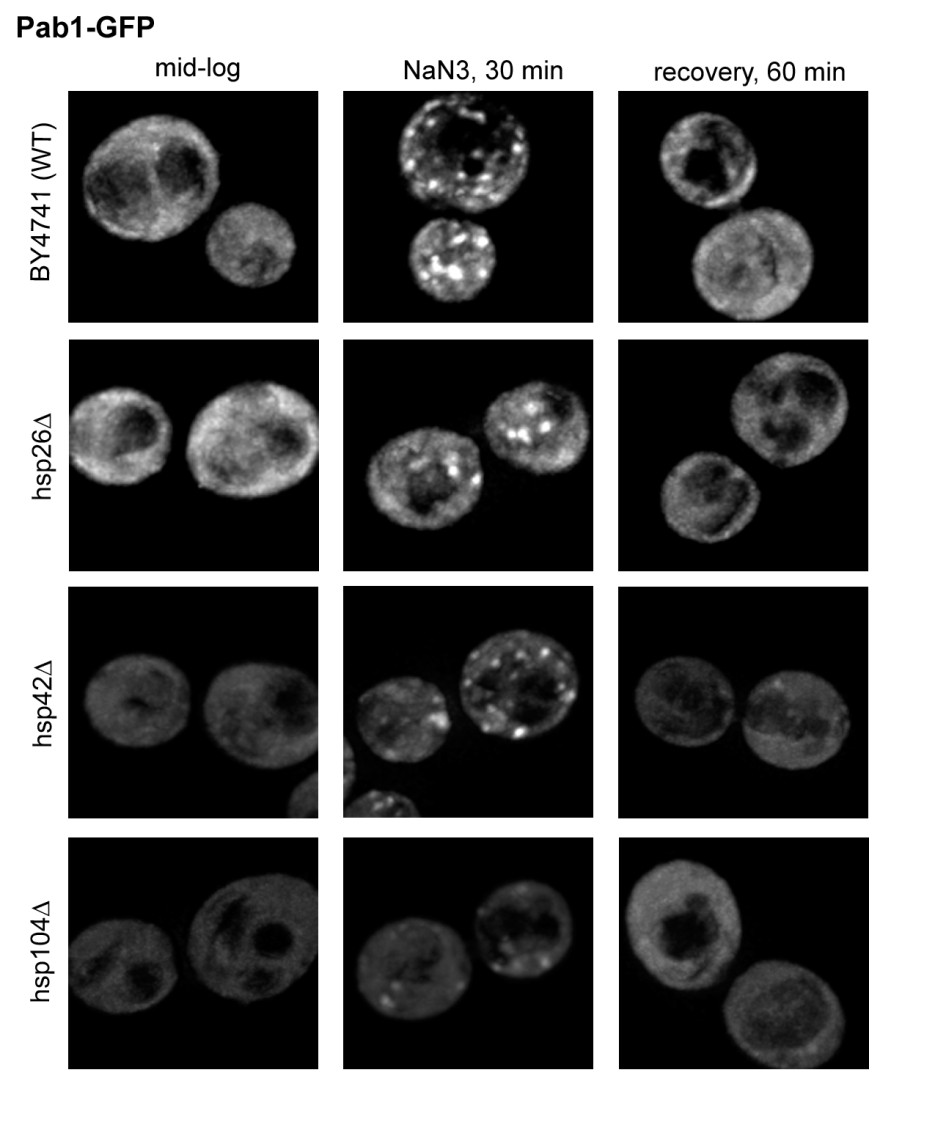


**FIGURE S2.**


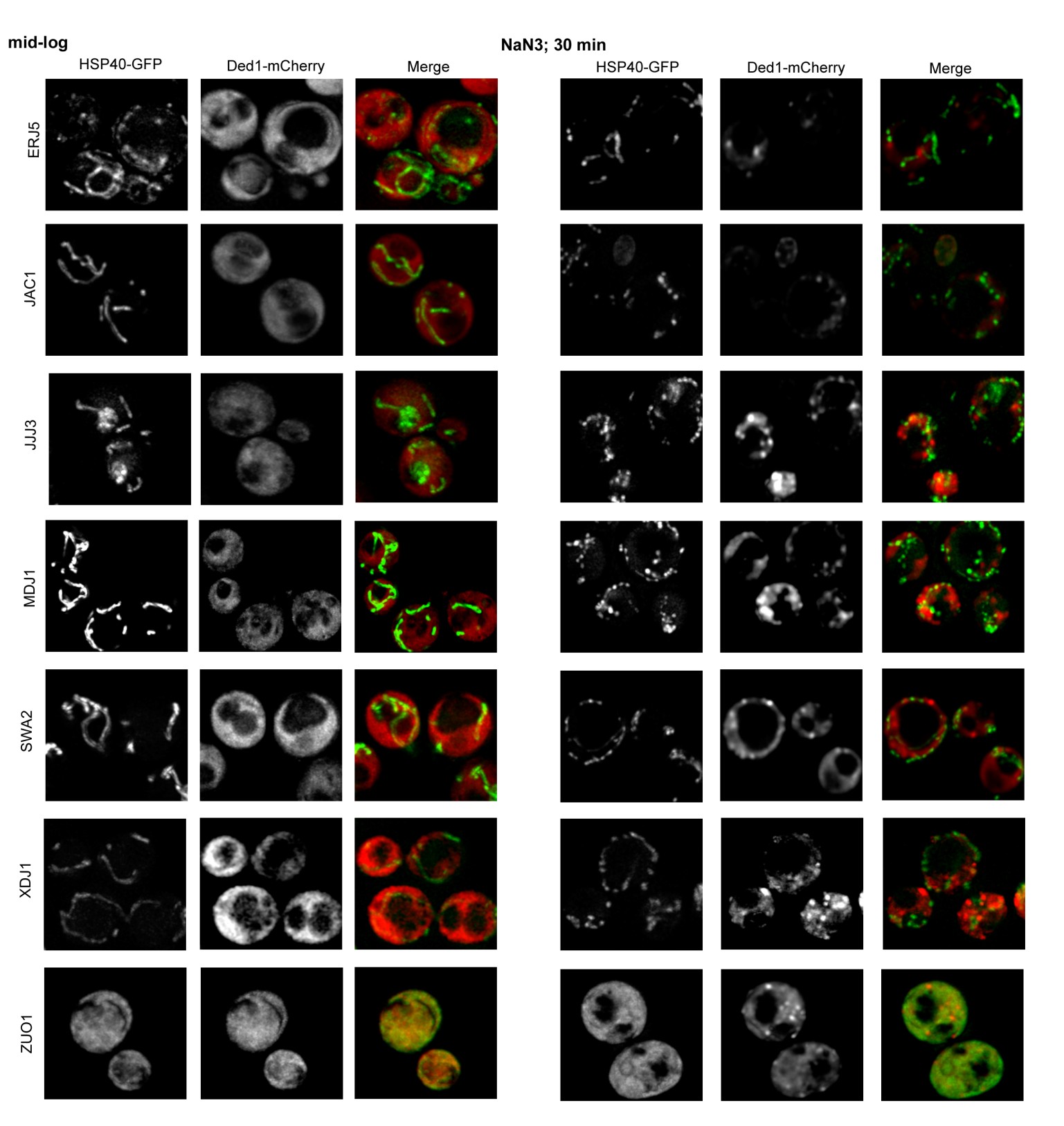


**Figure S1.** Analysis of stress granules in strains with deletions in individual heat shock proteins. Indicated strains were transformed with Pab1-GFP and stress granules examined during NaN3 stress and recovery. Growth conditions and recovery were carried out as in previous experiments.

**Figure S2.** Assessment of HSP40 protein co-localization with Ded1-mCherry. Indicated HSP40

GFP strains (from the yeast GFP collection) were transformed with Ded1-mCherry. GFP and mCherry cellular localization was then analyzed during exponential growth (mid-log) and after stress granule induction with NaN3 treatment.

**TABLE S1.**

| **Strains** | **Background** | **Mating type** | **Genotype** | **Reference** |
| --- | --- | --- | --- | --- |
| yRP2908 | DS10 | α | *his3-11, 3-15 leu2-3, 2-112, ura3-52, trp1D1 lys2* | Becker et al.,  1996 |
| yRP2909 | DS10 | α | *his3-11, 3-15 leu2-3, 2-112, ura3-52, trp1D1 lys2 ssa1-45 BKD ssa2-1 ssas3-1 ssa4-2 (ssa2:LEU2 ssa3:TRP1 ssa4:LYS2)* | Becker et al.,  1996 |
| yRP2912 | DS10 | a | *GAL2 his3-11, 15 leu2-3, 112 lys1 lys2 trp1 ura3-52 ydj1:HIS3* | Becker et al.,  1996 |
| yRP2914 | DS10 | α | *his3-11, 3-15 leu2-3, 2-112 ura3-52 trp1-D1 lys2 SSA1 ssa2-1 ssa3-1 ssa4-2 (ssa2:LEU2 ssa3:TRP1 ssa4:LYS2)* | Taxis et al., 2003 |
| yRP2961 | R1158 | a | *his3-1 leu2-0 met15-0 URA3::CMV-tTa pSIS1::Kan-tet-off TATA* | OPEN Biosystems |
| yRP2990 | JN516 | a | *his3-11, 15, leu2-3, 112, ura3-52, trp-D1, lys2*  *SSA1 (ssa2:LEU2 ssa3:TRP1 ssa4: LYS2)* | McClellan and  Brodsky, 2000 |
| yRP2991 | JN516 | a | *his3-11, 15, leu2-3, 112, ura3-52, trp-D1, lys2*  *SSA1 (ssa2:LEU2 ssa3:TRP1 ssa4: LYS2)* | McClellan and  Brodsky, 2000 |
| yRP2992 | JN516 | a | *his3-11, 15, leu2-3, 112, ura3-52, trp-D1, lys2*  *SSA1 (ssa2:LEU2 ssa3:TRP1 ssa4: LYS2)* | McClellan and  Brodsky, 2000 |
| yRP2433 | R1158 | a | *his3, leu2, lys2, ura3:CMVtTa* | OPEN Biosystems |
| yRP2894 | 840 | a | *his3, leu2, lys2, ura3:CMVtTa* | Buchan et al.  2014 |
| yRP3017 | BY4741 | a | *his3, leu2, lys2, ura3:CMVtTa atg15:NEO* | this study |
| yRP3024 | BY4741 | a | *his3, leu2, ura3:CMVtTa pSIS1: Kan-tet-off*  *TATA atg15:HYGB* | this study |

**TABLE S2**.

| **Plasmid** |  | **Reference** |
| --- | --- | --- |
| pRP1657 | Edc3-mCherry; Pab1-GFP (URA) | Buchan et al., 2008 |
| pRP2152 | Edc3-mCherry; Pab1-GFP (LEU) | this study |
| pRP2132 | Ded1-mCherry (URA) | Hilliker et al., 2011 |
| pRP2529 | pRS426 (URA3) | McClellan and Brodsky, 2000 |
| pRP2530 | pRS426-GPD-(His)6-SSA1 (URA3) | McClellan and Brodsky, 2000 |
| pRP2531 | pRS426-GPD-(His)6-SSA1[G199D] (URA3) | McClellan and Brodsky, 2000 |
| pRP1657 | Edc3-mCherry; Pab1-GFP (URA) | Buchan et al., 2008 |
